# Incomplete Concordance Between Host Phylogeny and Gut Microbial Community in Tibetan Wetland Birds

**DOI:** 10.3389/fmicb.2022.848906

**Published:** 2022-05-19

**Authors:** Tingbei Bo, Gang Song, Shiyu Tang, Mengru Zhang, Zhiwei Ma, Hongrui Lv, Yun Wu, Dezhi Zhang, Le Yang, Dehua Wang, Fumin Lei

**Affiliations:** ^1^State Key Laboratory of Integrated Management of Pest Insects and Rodents, Institute of Zoology, Chinese Academy of Sciences, Beijing, China; ^2^CAS Center for Excellence in Biotic Interactions, University of Chinese Academy of Sciences, Beijing, China; ^3^Key Laboratory of Zoological Systematics and Evolution, Institute of Zoology, Chinese Academy of Sciences, Beijing, China; ^4^University of Chinese Academy of Sciences, Beijing, China; ^5^School of Ecology and Environment, Anhui Normal University, Wuhu, China; ^6^Tibet Plateau Institute of Biology, Lhasa, China; ^7^School of Life Sciences, Shandong University, Qingdao, China

**Keywords:** birds, gut microbiota, Tibetan Plateau, phylogeny, adaptation

## Abstract

Gut microbial communities of animals play key roles in host evolution, while the relationship between gut microbiota and host evolution in Tibetan birds remains unknown. Herein, we sequenced the gut microbiota of 67 wild birds of seven species dwelling in the Tibetan wetlands. We found an obvious species-specific structure of gut microbiota among these plateau birds whose habitats were overlapped. Different from plateau mammals, there was no strict synergy between the hierarchical tree of gut microbial community and species phylogeny. In brown-headed gulls (*Larus brunnicephalus*) as an example, the structure of gut microbiota differed in different habitats, and the relative abundance of bacteria, such as *Lactobacillus*, *Streptococcus, Paracoccus, Lachnospiraceae*, and *Vibrio*, significantly correlated with altitude. Finally, we found various pathogenic bacteria in the birds of these plateau wetlands, and the interspecific differences were related to their diet and living environments.

## Introduction

Previous studies have revealed that gut microbiota plays crucial roles in many physiological processes of vertebrates, including intestinal morphology, digestive functions, and immune health (Backhed et al., 2005). McFall-Ngai et al. (2013) proposed that animals and microbes could be co-evolving together. For example, in African ape species, the dissimilarity between gut microbial communities is correlated with the evolutionary distance between the host species (Moeller et al., 2014). Studies on eight small mammal species distributed in the Qinghai-Tibet Plateau (QTP) and Inner Mongolia grassland show that the gut microbial composition is associated with the host phylogeny (Li et al., 2017).

Birds are a group of warm-blooded vertebrates that show strong adaptability to the environmental changes and are characterized by complex life history and diversified feeding habits and migration patterns (Kohl, 2012). Previous work has revealed that the taxonomic composition of a bird’s microbiome differs with the host species (Waite and Taylor, 2014; Hird et al., 2015), and a high variation has also been observed within the species (Hird et al., 2014). One study on woodlarks (*Lullula arborea*) and skylarks (*Alauda arvensis*) shows that niche environment plays a stronger role in shaping the composition of bird gut microbiota than phylogeny (Veelen et al., 2017). The Tibetan Plateau is well known for its extreme environmental conditions, such as extreme cold, strong UV light, and low oxygen levels, which pose a challenge to the survival and reproduction of birds. Although there are intensive studies on the evolutionary history and adaptation of birds in QTP (Qu et al., 2013, 2020, 2021; Zhu et al., 2018), little is known about the gut microbial ecology of such high-plateau birds and the relationship between host phylogeny and gut microbiota.

In the present study, we selected seven species of birds that are commonly encountered in the plateau wetlands of north Tibet during the breeding season. Bar-headed goose (*Anser indicus*), ruddy shelduck (*Tadorna ferruginea*), and brown-headed gull (*Larus brunnicephalus*) are commonly found in large areas of Northwest China, Mongolia, Kazakhstan, and Russia (Liu and Chen, 2021), while they are the dominant species in plenty of water bodies located in north Tibet during the summer season. The common redshank (*Tringa totanus*) and lesser sand plover (*Charadrius mongolus*) are two waders of smaller body size often found in shallow water bodies, such as lake coasts and riversides. The hill pigeon (*Columba rupestris*) inhabits relatively drier environments and rests on rock reefs, whereas the plain-backed snowfinch (*Pyrgilauda blanfordi*) dwells on highland meadows and lakeside grasslands. These species belong to four avian orders (Anseriformes, Charaderiiformes, Columbiformes, and Passeriformes), representing the various and typical ecological groups of birds inhabiting high-altitude wetlands of north Tibet. Therefore, we studied the gut microbiota of these target species to evaluate the characteristics of the gut microbiota and assess the relationship between host phylogeny and gut microbiota. In this context, we propose to get clues from the gut microbiome that facilitate the adaptation of wild birds to high altitudes.

## Materials and Methods

### Sample Collection

As most of the birds migrate to distant places to escape from extremely harsh conditions during the winters, we carried out field trips and sampling work from 20 July to 15 August, 2021, the time corresponding to the late breeding season of birds in the north Tibet. We found that all these species except for plain-backed snowfinch perched and always moved in flocks. We observed the bird flock for more than 30 min, identified the species, and counted their number by viewing through 8 × 42 binoculars and a 25 × 60 spotting scope. Then, fresh fecal samples were collected from the spots where the flocks rested. We captured the plain-backed snowfinch using mist nets. The trapped birds were removed into clean bird bags independently for 2 h. Then the fecal samples were collected, and the birds were released after the morphology measurements using a clipper. The samples were placed in 2-ml sterilized storage tubes and stored in liquid nitrogen immediately. After the field trip, the samples were immersed in dried ice during transportation and later stored in a lab freezer at −80^°^C.

### 16S rRNA Gene Sequencing Analysis

The DNA was extracted from the fecal samples by using the QIAamp DNA stool Mini Kit from Qiagen (Germany), according to the manufacturer’s instructions. The 16S rRNA gene, comprising V3 and V4 regions, was amplified by PCR using composite-specific bacterial primers (338F 5′-ACTCCTACGGG AGGCAGCA-3′; 806R 5′-GGACTACHVGGGTWTCTAAT-3′). High-throughput pyrosequencing of the PCR products was performed on an Illumina MiSeq platform. The raw paired-end reads from the original DNA fragments were merged using FLASH32 and assigned to each sample according to the unique barcodes. For the bioinformatics analysis, high-quality reads were performed, and all the effective reads from each sample were clustered into operational taxonomic units (OTUs) based on 97% sequence similarity according to UCLUST33. For alpha diversity analysis, we rarified the OTUs to several metrics, including the Shannon index. For beta diversity analysis, principal coordinate analysis (PCoA) was performed using Bray–Curtis and weighted UniFrac distance matrices. The method of unweighted pair group method with arithmetic mean (UPGMA) was used to establish the phylogenetic trees for bacteria and judge the differences between the samples. The LDA effect size (LEfSe) analysis was performed for the quantitative analysis of biomarkers within each group. Briefly, LEfSe analysis, LDA threshold of > 3, used the non-parametric factorial Kruskal–Wallis (KW) sum rank test.

### Host Phylogeny Extraction and Statistical Analysis

TimeTree^1^ was used to build a phylogenetic tree with a timeline for these seven species of birds (Hedges et al., 2006; Kumar et al., 2017). TimeTree is a public knowledge base that provides information on the evolutionary timescale of life. Data from thousands of published studies are assembled into a searchable tree of life scaled to time. All prinicpal coordinate analysis (PCoA) were based on Bray–Curtis and weighted UniFrac distances using evenly sampled OTU abundances. Significance for PCoA (β-diversity) analyses was checked using PERMANOVA. The Bray–Curtis dissimilarity metrics were compared with the distance matrix of the host branch length using Mantel’s test to test the correlation between the host phylogeny and gut microbiota. The microbial tree was built by UPGMA based on all the samples collected from seven species of birds. The correlation between microbiome and environmental factors was analyzed by Spearman correlation. Differences between groups were statistically analyzed using KW test with a level of *P* < 0.05 (*P < 0.05, ^**^P < 0.01, ^***^P < 0.001).

## Results

**FIGURE 1:**
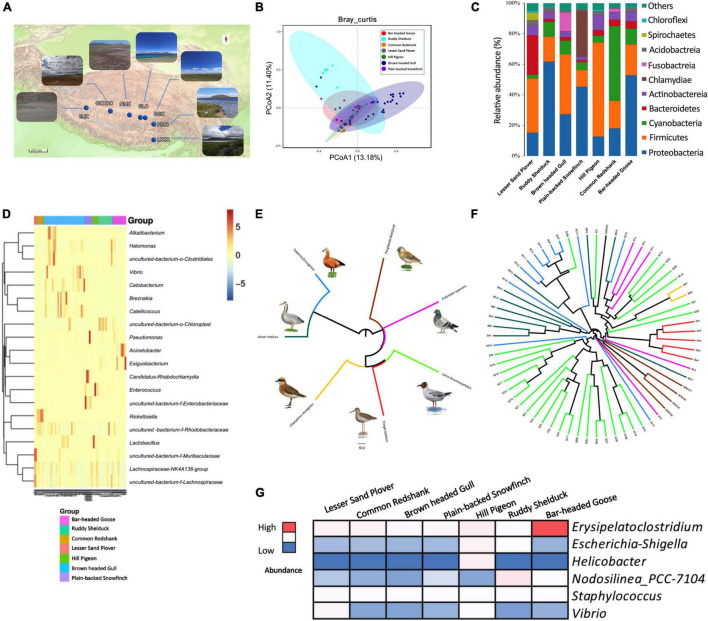
Gut microbial diversity of birds and phylogenetic tree based on variations in complete mitochondrial gene sequences and composition of the gut microbiome. **(A)** Sampling location and photo record of habitats. **(B)** PCoA plots of Bray–Curtis distance for 67 birds. **(C)** Result of gut microbiota in seven species of birds at the phylum level. **(D)** Heat map of the microbial composition of seven species of birds at the genus level. **(E)** Host phylogeny based on mitochondrial genes. **(F)** Gut microbiome phylogeny based on Bray–Curtis dissimilarity. Branches of the same color represent the same species. The closer the samples, the shorter the branch length, indicating that the species composition of the two samples is more similar. **(G)** Differences in the abundance of pathogens among different species.

We sequenced the V3 and V4 regions of bacterial 16S rRNA genes of 67 fecal samples from seven species of birds: bar-headed goose (*Anser indicus*), brown-headed gull (*Larus brunnicephalus*), ruddy shelduck (*Tadorna ferruginea*), lesser sand plover (*Charadrius mongolus*), common redshank (*Tringa totanus*), hill pigeon (*Columba rupestris*), and plain-backed snowfinch (*Pyrgilauda blanfordi*). The sampling work was carried out at five localities of the Tibet Autonomous Region in summer of 2021, and the regions from west to east were as follows: GJX, GZXDC, NMX, SLC, BGX, NMC, and LKZX (Supplementary Tables 1, 2 and Figure 1A). In PCoA analysis, the gut microbial assemblages were clustered by Bray–Curtis and weighted UniFrac distance matrices (PERMANOVA, *P* < 0.05, Figure 1B and Supplementary Figure 1A). Microbial composition among groups overlapped across most host species at the phylum level, but differences were observed among Firmicutes, Bacteroidetes, Cyanobacteria, and Proteobacteria (Figure 1C). For instance, Proteobacteria were abundant in bar-headed goose and ruddy shelduck, Bacteroidetes were abundant in lesser sand plover, and Cyanobacteria were abundant in common redshank (Figure 1C). At the genus level, the microbiota of brown-headed gull was mainly dominated by *Breznakia*, *Cetobacterium, Vibrio, Alkalibacterium*, and *Halomonas*. *Rickettsiella* was higher in common redshank, *Lactobacillus* and *Enterococcus* were higher in hill pigeons, and *Acinetobacter* and *Exiguobacterium* were predominant in bar-headed goose (Figure 1D). The results of PICRUSt revealed higher stability in functions, in general, with respect to the variability seen at the taxonomic level. The LEfSe analysis showed statistically significant differences in the lipid metabolism, energy metabolism, metabolism of co-factors, and vitamins at level 2 in the KEGG hierarchy (Supplementary Figure 1B). The Mantel test between the distance metrics of host genes and Bray–Curtis dissimilarity showed that there was no significant overall correlation between host phylogeny and gut microbiota (*r* = 0.1018, *P* = 0.2). Our data showed incomplete concordance between host phylogeny and gut microbial communities in the Tibetan wetland birds (Figures 1E,F). At the genus level, we also observed several common potentially pathogenic bacteria, including *Erysipelatoclostridium, Escherichia-Shigella, Helicobacter, Nodosilinea_PCC-7104, Staphylococcus*, and *Vibrio. Erysipelatoclostridium* accounted for a relatively high proportion of bacteria, particularly in bar-headed gooses. *Helicobacter* was found to be abundant in hill pigeons (Figure 1G).

**FIGURE 2:**
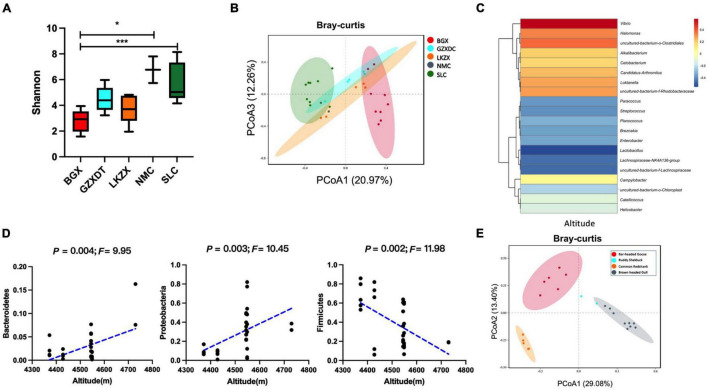
Gut microbial diversity of brown-headed gulls from five habitats and gut microbial diversity of four species of birds in BGX. **(A)** The Shannon index. **(B)** PCoA plots of Bray–Curtis distance. **(C)** Correlation between three phyla and altitude (Spearman). **(D)** Heat map of correlation between microbial composition and altitude at the genus level. **(E)** PCoA plots of Bray–Curtis distance of four species in BGX. Data indicate mean ± SEM values. *P < 0.05 and ***P < 0.001.

To further explore the impact of habitat differences, we compared the gut microbiota diversity of brown-headed gulls inhabiting five geographical regions. Alpha diversity was higher in the SLC and NMC (Figure 2A). In PCoA analysis, most of the gut microbial assemblages clustered by locality (PERMANOVA, *P* = 0.001, Figure 2B and Supplementary Figure 1C). There were differences in the proportion of Firmicutes, Cyanobacteria, and Proteobacteria among the five localities (Supplementary Figure 1D). UPGMA clustering showed that microbial clustering was not entirely consistent within the localities, which may be attributed to individual dispersal due to migration (Supplementary Figure 1E). Microbial composition was closely related to habitat, for example, Bacteroidetes and Proteobacteria were positively correlated with altitude, while Firmicutes were negatively correlated with altitude (Figure 2D). *Lactobacillus, Streptococcus, Paracoccus*, and *Lachnospiraceae* populations were negatively correlated with altitude, while *Vibrio* was positively correlated with altitude (Figure 2C and Supplementary Table 3). To explore the impact of bird species on microbial diversity, we compared the four water species (bar-headed goose, brown-headed gull, ruddy shelduck, and common redshank) distributed in the same habitat, BGX. The results of PCoA showed that although the birds lived in the same area, significant differences in the gut microbiota were observed among the species (PERMANOVA, *P* < 0.001, Figure 2E and Supplementary Figure 1F). The microbial composition and proportion were also different at the genus level (Supplementary Figure 1G).

## Discussion

This is the first study to investigate the composition and diversity of the gut microbiota in the wild birds dominating high-plateau regions and determine the relationship between host phylogeny and their gut microbiota. Our results indicate that the impact of environmental factors on the gut microbiota of plateau birds would be the main factor rather than phylogeny. Therefore, we should carefully interpret the information about gut microbiota while inferring the evolution of plateau birds.

### Features of Gut Microbiota of Plateau Birds

The results of our study indicate that the gut microbiota of the plateau birds is dominated by Firmicutes, Cyanobacteria, and Proteobacteria. The characteristic feature of Firmicutes is the synthesis of short-chain fatty acids (SCFAs), which are found to be higher in other plateau endemic animals, like plateau yaks (*Bos mutus*) (Zhang et al., 2016) and plateau pika (*Ochotona curzoniae*) (Li et al., 2016). Higher SCFA content can satisfy the physiological or energy demands of the host in cold and hypoxic high-altitude environments. Although living in the same extreme environments of the Qinghai-Tibet Plateau, the seven species of birds show differences in the composition of the microbiota. The different levels of gut microbial diversity between the host species may be partly explained by the differences in diet quality. For example, bar-headed goose and ruddy shelduck mainly feed on invertebrates and plants, while the hill pigeon and plain-backed snowfinch feed on seeds and insects. Although bar-headed goose and ruddy shelduck have similar gut microbiota and diet, the synthetic pathways related to functional adaptation might be different. For instance, bar-headed geese have more gut bacteria related to amino acid metabolism and lipid metabolism, while the proportion of bacteria involved in energy, co-factor, and vitamin metabolism is higher in ruddy shelduck. This finding reflects the species specificity of gut microbiota and ecological niche differentiation between the two species. The common redshank and lesser sand plover are two small-sized waders often found together in shallow water bodies. Their habitats and feeding habits are similar, but the structure of gut microbiota is not similar. Bacteroidetes are abundant in lesser sand plover, while Cyanobacteria are abundant in common redshank. We also noticed that different birds harbor different types of potentially pathogenic microbiota. We observed that the feces of bar-headed goose contains a large proportion of *Erysipelatoclostridium*, which may be due to the pollution of the water in which they live or contact with the pathogenic bacteria in water. Hill pigeons usually live closely with humans, so they carry more number of *Escherichia-Shigella* and *Helicobacter* species in their feces. Therefore, harboring and transmission of potentially pathogenic bacteria by the wild birds of the plateau is a topic that is worthy of further study in the future.

### Relationship Between Host Species Phylogeny and Their Gut Microbiota

Previous studies on mammals show that various internal and external factors influence the composition and diversity of the gut microbiota, including host phylogeny, gut morphology, diet, physiological status, geography, and body weight (Ley et al., 2006, 2008; Santacruz et al., 2010; Linnenbrink et al., 2013). Therefore, co-evolution between host and microbial lineages has played a key role in the adaptation of mammals to their diverse lifestyles. Most mammalian microbiomes are strongly correlated with host phylogeny (Ley et al., 2008). Findings in other vertebrates also showed that closely related lineages harbor more similar gut microbiota than distantly related lineages (Ochman et al., 2010; Sanders et al., 2014). As for plateau animals, for example, Pika species, the host relationship affects the structure of the gut microbiota apparently, showing a significant overall correlation between host phylogeny and gut microbiota (Li et al., 2017). However, in birds, only a few studies have found a correlation between the gut microbiome and host phylogeny. For example, studies on four waterbird species in Israel, great cormorant (*Phalacrocorax carbo*), little egret (*Egretta garzetta*) black-crowned night heron (*Nycticorax nycticorax*), and black-headed gull (*Larus ridibundus*), suggest a correlation between the host phylogeny and their intestinal microbial community hierarchical tree, thus displaying phylosymbiosis (Laviad-Shitrit et al., 2019). A study including 491 species of birds showed a weak correlation between host factors and microbiome composition, while most of their intestinal bacteria exhibited no host specificity (Song et al., 2020). Our results show that the phylogeny of birds does not influence their intestinal microbial community hierarchical tree in Tibet wetlands, which is inconsistent with the existing research on plateau mammals and may reflect a unique feature of high-plateau birds.

### Altitude Affects the Composition of Gut Microbiota

Previous research has shown that the avian microbiomes have relatively low stability (Song et al., 2020; Bodawatta et al., 2021) and high malleability to environmental and dietary changes (Bodawatta et al., 2021). Similarly, our results show that brown-headed gulls from different localities have different intestinal microbial structures and that the gut microbiome of these birds inhabiting the same habitat is more similar. The study on woodlarks and skylarks has also shown that sharing an ecological niche among hosts (either species or individuals) leads to the convergence of their microbiota (Veelen et al., 2017). Through correlation analysis, we found that altitude is the main factor that affects the proportion of key bacteria. With an increase in altitude, the proportion of Firmicutes, including *Lactobacillus* and *Lachnospiraceae*, in brown-headed gulls decreases. *Lactobacillus* play major roles in the SCFAs (Borda-Molina et al., 2018), food digestion, and energy conversion. Lachnospiraceae include obligate anaerobic bacteria that affect the host health by producing short-chain fatty acids, participating in bile acid metabolism, and promoting colonization resistance to intestinal pathogens (Sorbara et al., 2020; Xu et al., 2021). Therefore, our results imply that low-altitude brown-headed gulls may rely on the relative abundance of *Lactobacillus* and *Streptococcus* to adapt to the complex food and pathogen-rich environment. At higher altitudes, the proportion of Bacteroidetes and Proteobacteria is increased, including *Vibrio* and *Paracoccus*, which may be attributed to the weakening of immune function, suggesting that the gut microbiome may be involved in regulating the immune functions of high-altitude birds (Qu et al., 2013). However, microbial clustering is not completely consistent with geographical separation, which suggests that there might be some individual migrants and shared microbiome across different localities in brown-headed gulls.

## Conclusion

Although the composition of microbiomes is different among species, our results show that the gut microbial community is not in line with the phylogenetic relationship in Tibet wetland birds. Environmental factors, such as altitude, are the key factors that affect the gut microbiota of plateau birds. Our results provide a new idea for exploring the relationship between gut microbiota and the evolution and adaptability of high-plateau animals, and suggest that one should be cautious while taking into account the gut microbiota to infer the evolution pattern in plateau birds.

## Data Availability Statement

Raw sequence data are deposited in the NCBI Sequence Read Archive under accession BioProject PRJNA787368.

## Author Contributions

ZM, HL, YW, DZ, and LY: field work and experiment. TB, GS, ST, MZ, and DW: writing. DW and FL: supervision and project management. TB, GS, and FL: funding acquisition. TB and GS: data analysis. All authors contributed to the article and approved the submitted version.

## Conflict of Interest

The authors declare that the research was conducted in the absence of any commercial or financial relationships that could be construed as a potential conflict of interest.

## Publisher’s Note

All claims expressed in this article are solely those of the authors and do not necessarily represent those of their affiliated organizations, or those of the publisher, the editors and the reviewers. Any product that may be evaluated in this article, or claim that may be made by its manufacturer, is not guaranteed or endorsed by the publisher.

## Footnotes

1
http://www.timetree.org
