# Supplementary material for: Incomplete Concordance Between Host Phylogeny and Gut Microbial Community in Tibetan Wetland Birds
Source: Front Microbiol. 2022 May 19;13:848906. doi: 10.3389/fmicb.2022.848906 (PMC9161150; doi:10.3389/fmicb.2022.848906)
Supplement: Supplementary file 1 [file Data_Sheet_1.docx]

**Supplementary**

Supplementary Figure 1


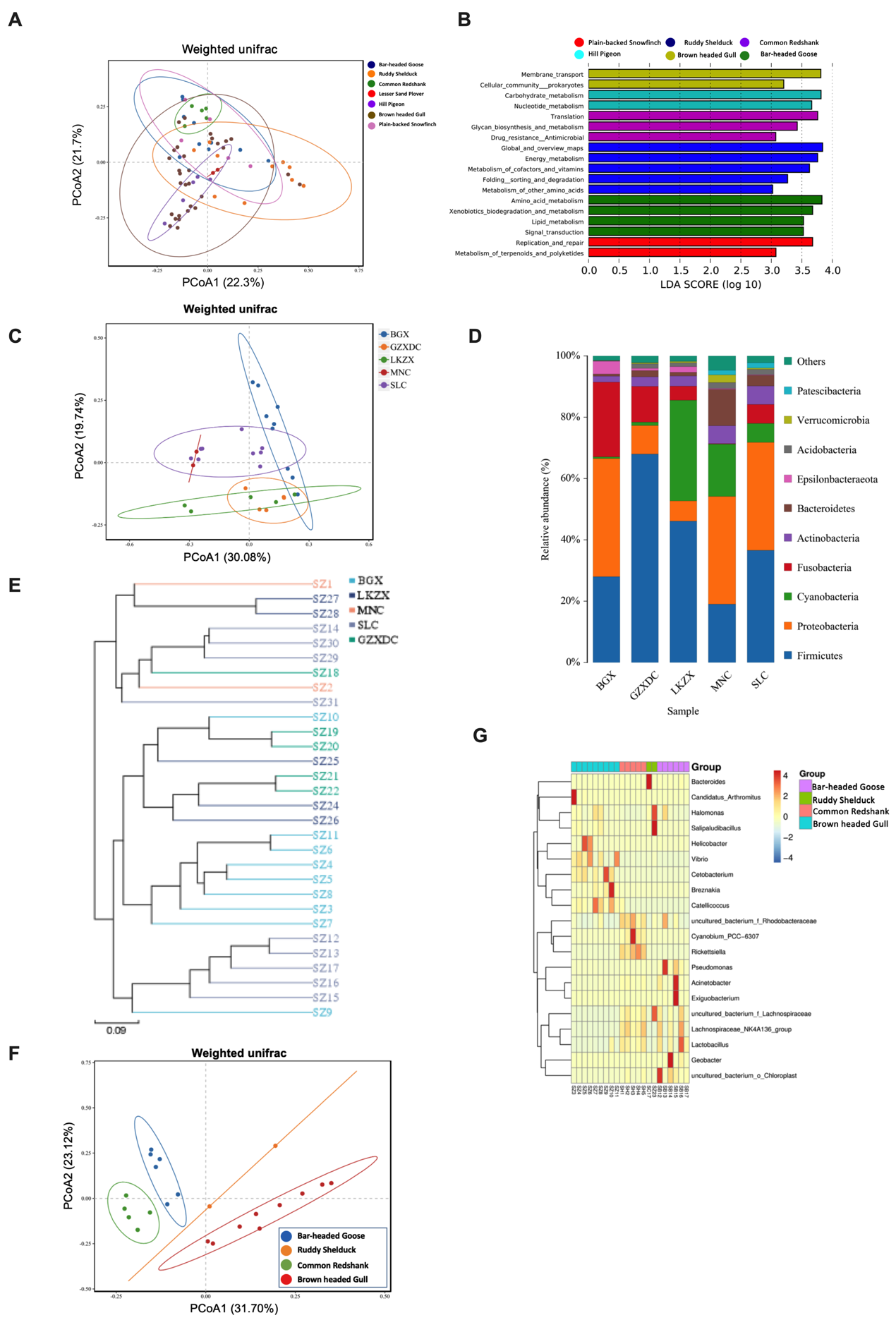


Supplementary results. (A) PCoA plots of Weighted unifrac distance for 67 birds. (B) Results from LEfSe analysis, showing the most differentially abundant function enriched from 7 species of birds. (C) PCoA plots of Weighted unifrac distance for Brown headed gulls from five localities. (D)Result of phylum level of gut microbiota in Brown headed gulls from five localities. (E) UPGMA clustering showed that microbial clustering was not consistent with localities. (F) PCoA plots of Weighted unifrac distance for of 4 species in BGX. (G) Heat map of microbial composition at genus level of 4 species in BGX.

Supplementary Table 1

| Species | Family | Diet | Habitat | n |
| --- | --- | --- | --- | --- |
| Bar-headed Goose  (*Anser indicus*) | *Anseridae* | Invertebrates, plants | NMC | 4 |
|  |  |  | BGX | 6 |
| Ruddy Shelduck (*Tadorna ferruginea*) | *Anseridae* | Invertebrates, plants | NMX | 6 |
|  |  |  | GZXDC | 2 |
|  |  |  | BGX | 2 |
| Common Redshank  (*Tringa totanus*) | *Scolopacidae* | Invertebrates, insects,  small fish | BGX | 5 |
| Lesser Sand Plover  (*Charadrius mongolus*) | *Charadriidae* | Invertebrates, insects | NMC | 2 |
| Hill Pigeon  (Columba rupestris) | *Columbidae* | Fruits, plants, seeds, grains | GJX | 5 |
| Brown headed Gull (*Larus brunnicephalus*) | *Laridae* | Invertebrates, insects | NMC | 2 |
|  |  |  | BGX | 9 |
|  |  |  | SLC | 9 |
|  |  |  | GZXDC | 5 |
|  |  |  | LKZX | 5 |
| Plain-backed Snowfinch  (*Pyrgilauda blanfordi*) | *Passeridae* | Insects, plants, seeds | NMC | 5 |

Supplementary Table2

| Habitat | Altitude(m) | Longitude and latitude |
| --- | --- | --- |
| NMX | 4496 | 31.82；87.48 |
| GZXDC | 4372 | 32.20；84.68 |
| NMC | 4730 | 30.77；90.96 |
| SLC | 4545 | 31.52；89.08 |
| LKZX | 4428 | 28.91；90.94 |
| BGX | 4591 | 31.53；89.74 |
| GJX | 4470 | 32.42；81.03 |

Supplementary Table 3. Correlation between genus and Altitude in Brown headed gulls.

| Genus | P value |
| --- | --- |
| *Alkalibacterium* | 0.277828576 |
| *Breznakia* | 0.056408434 |
| *Campylobacter* | 0.730969714 |
| *Candidatus_Arthromitus* | 0.204607356 |
| *Catellicoccus* | 0.531033727 |
| *Cetobacterium* | 0.358621933 |
| *Enterobacter* | 0.059785344 |
| *Halomonas* | 0.078693409 |
| *Helicobacter* | 0.489529296 |
| *Lachnospiraceae_NK4A136_group* | 0.011873196 |
| *Lactobacillus* | 0.001525888 |
| *Loktanella* | 0.158249009 |
| *Paracoccus* | 0.043087155 |
| *Planococcus* | 0.085606664 |
| *Streptococcus* | 0.035366762 |
| *Vibrio* | 0.000755647 |
| *uncultured_bacterium_f_Lachnospiraceae* | 0.00902278 |
| *uncultured_bacterium_f_Rhodobacteraceae* | 0.130880537 |
| *uncultured_bacterium_o_Chloroplast* | 0.214409251 |
| *uncultured_bacterium_o_Clostridiales* | 0.042809776 |
